# *CTM4DOC*: electronic structure analysis from X-ray spectroscopy

**DOI:** 10.1107/S1600577516012443

**Published:** 2016-08-23

**Authors:** Mario Ulises Delgado-Jaime, Kaili Zhang, Josh Vura-Weis, Frank M. F. de Groot

**Affiliations:** a Inorganic Chemistry and Catalysis Group, Debye Institute for Nanomaterials Science, Utrecht University, Universiteitsweg 99, Utrecht 3584 CG, The Netherlands; b Department of Chemistry, University of Illinois at Urbana-Champaign, Urbana, IL 61801, USA

**Keywords:** multiplet simulations, electronic structure, differential orbital covalency, X-ray spectroscopy

## Abstract

Two electronic structure descriptions, one based on orbitals and the other based on term symbols, have been implemented in a new Matlab-based program, *CTM4DOC*. The program includes a graphical user interface that allows the user to explore the dependence of details of electronic structure in transition metal systems, both in the ground and core-hole excited states, on intra-atomic electron–electron, crystal-field and charge-transfer interactions. The program can also track the evolution of electronic structure features as the crystal-field parameters are systematically varied, generating Tanabe–Sugano-type diagrams. Examples on first-row transition metal systems are presented and the implications on the interpretation of X-ray spectra and on the understanding of low-spin, high-spin and mixed-spin systems are discussed.

## Abstract 2

*CTM4DOC*, a new graphical user interface for electronic structure calculations in X-ray spectroscopy, is introduced and examples in transition metal complexes are described.

## 1. Introduction   

*L*-edge X-ray spectroscopy is a powerful method for the determination of the electronic and magnetic structure of transition metal ions in molecules and solids. The shapes of *L*-edge X-ray absorption spectra are dominated by the excitation of 2*p* electrons to empty 3*d* states and can be accurately modelled and interpreted using crystal-field multiplet theory applied to transitions from a 3*d* ^*N*^ ground-state configuration to a 2*p* ^5^3*d* ^*N*+1^ final-state configuration. This crystal-field multiplet model has been developed by Thole and co-workers (Butler, 1981 ▸; Cowan, 1981 ▸; de Groot *et al.*, 1990 ▸; de Groot & Kotani, 2008 ▸; Thole *et al.*, 1988 ▸) and forms the basis of the *CTM4XAS* interface (Stavitski & de Groot, 2010 ▸). Systems that have increased covalency cannot be accurately described by crystal-field theory and require the inclusion of charge-transfer channels in their description. In molecular systems, charge transfer can be strongly angular-dependent, an observation that led to the development of differential orbital covalency (DOC) simulations, originally applied to inorganic and bio-inorganic systems (Hocking *et al.*, 2006 ▸, 2007 ▸, 2009 ▸, 2010 ▸; Lundberg *et al.*, 2013 ▸; Wasinger *et al.*, 2003 ▸). Recently, a number of first-principle routes have been developed for solid-state-based methods by the groups of Haverkort (Haverkort *et al.*, 2012 ▸, 2014 ▸), Hariki (Hariki *et al.*, 2013 ▸, 2015 ▸) and Ikeno (Ikeno *et al.*, 2011 ▸). In the case of molecules, first-principle methods are mainly based on restricted active space and they have been developed in the groups of Neese (Roemelt *et al.*, 2013 ▸; Maganas *et al.*, 2013 ▸), Lundberg (Lundberg *et al.*, 2013 ▸) and Odelius (Pinjari *et al.*, 2014 ▸).

Multiplet analysis is applied in many experimental studies. It is typically used to determine the valence, the spin state and the crystal-field parameters, by tuning the parameters to optimize the fit with experimental spectra. In a covalent system the DOC is also determined. The electronic states of a transition metal ion in a cubic crystal field can be defined in a variety of descriptions. In an orbital description (strong-field representation), the electronic state is described in terms of occupation numbers of *t*_2*g*_ or *e*_*g*_ orbitals. Under such a description, the ground state of a high-spin 3*d* ^5^ system, for instance, can be described as 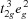. However, the electronic structure of an open-shell system is dominated by strong electron–electron interactions, resulting in non-integral occupation of one-electron valence orbitals. In such a system, a more convenient alternative description involves expressing electronic states in terms of their atomic components. This weak-field description shows the relative contributions of atomic term symbols in the ground state. In most cases the term symbol notation in the point group symmetry of the transition metal ion is most useful as it combines the atomic two-electron integrals with cubic crystal-field effects. It is important to note here that the term symbol description is only a label and an electronic state usually must be described as a linear combination of term symbols, where the 3*d* spin–orbit coupling causes a mixture between different spin states.

**Figure 1:**
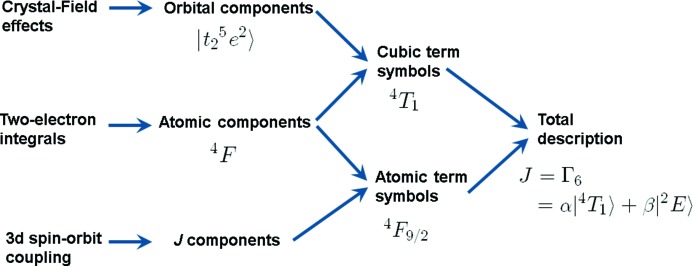
Interactions and associated symmetry labels relevant to the localized electronic structure of an isolated high-spin 3*d* ^7^ system. In cases where charge-transfer effects play an important role, this description would lead to the mixing of 3*d* ^7^ with 3*d* ^8^ *L* states.

Fig. 1 ▸ gives a systematic description of the main interactions and their consequences on symmetry, including the term symbol notations. If only the crystal field is included, the 3*d* orbitals are split into *t*_2*g*_ and *e*_*g*_ manifolds and the state can then be described just as a linear combination of orbital components. Using a 3*d* ^7^ high-spin system as an example in Fig. 1 ▸, the ground state is then described simply as *t* ^5^ *e* ^2^. The second interaction is given by two-electron integrals that give rise to the atomic components (^4^ *F*). Together, the crystal field and the two-electron integrals create the cubic term symbols (^4^ *T*_1_). The cubic term symbols can be decomposed into their (range of) orbital components or their corresponding atomic components.

The third interaction to consider in Fig. 1 ▸ is the 3*d* spin–orbit coupling, which splits the electronic states in terms of their total angular momentum (*J*) components. Combined with the two-electron integrals this yields the atomic term symbols. Combining all three interactions together yields the total term symbol *J* = Γ_6_ that can be decomposed into its cubic term symbol components, for example α|^4^ *T*_1_〉 + β|^2^ *E*〉. Alternatively, it can be decomposed into its atomic term symbols. Subsequently, the total term symbol can be developed into its orbital, atomic and *J* components.

The three interactions described above define one localized 3*d* ^*N*^ configuration. Inclusion of charge transfer causes this configuration to be mixed with a second configuration 3*d* ^*N*+1^ *L*. This second configuration itself can also be decomposed in an analogous manner as the first configuration. After inclusion of charge transfer, a given electronic state can be separated into its base and charge-transfer configuration and each of these configurations can be separated into their three specific components (orbital, atomic and *J* components), together yielding a picture of the *local* structure of a transition metal ion. In the general case, this picture must be extended with more ligand-to-metal charge-transfer configurations, metal-to-ligand charge-transfer configurations, *p*–*d* hybridizations and metal-to-metal charge-transfer configurations. In solids, translational symmetry will modify this picture further.

**Figure 2:**
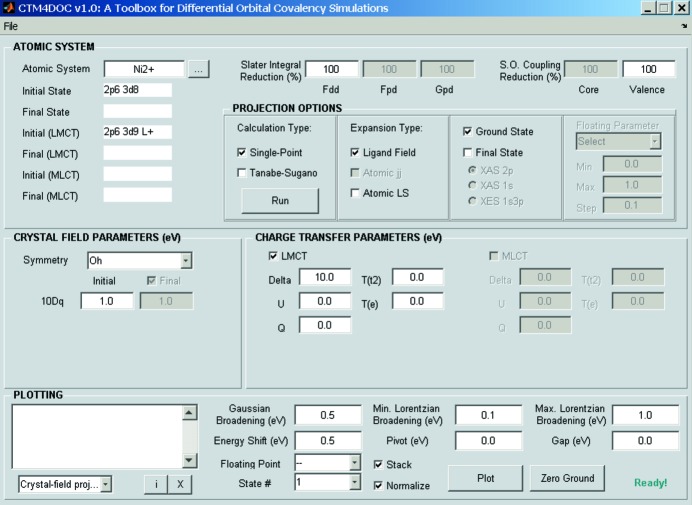
Screenshot of the graphical user interface of *CTM4DOC*.

To represent the ground and excited states in terms of any of the above-mentioned descriptions, a projection method first reported in 2003 is utilized in *CTM4DOC* (Fig. 2 ▸), a new Matlab-based program where three descriptions are implemented to characterize electronic states of transition metal complexes: one that uses a linear combination of atomic term symbols; another one that uses a linear combination of crystal-field term symbols of cubic symmetry; or, for an orbital description, a strong-field representation defined as linear combinations of crystal-field configurations,
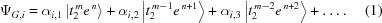
To calculate the coefficients α_*ij*_ in this expansion, this projection method involved a dummy 1*s*-to-4*p* transition (both being spectator orbitals decoupled from the 2*p*–3*d* system) between each of the ground-state multiplets (Ψ_*g*,*i*_) and each of the ideal crystal-field configurations, which were constructed by setting to zero all atomic parameters (Slater integrals and spin–orbit coupling parameters). Thus, the resulting calculated intensity obtained for each transition is directly proportional to the expansion coefficients, α_*ij*_. By representing the ground-state multiplets according to this expansion, the evaluation of metal-3*d* covalency (of *t*_2_ and *e* orbitals) allows the comparison with ground-state density functional theory (DFT) calculations. More recently (Kroll *et al.*, 2015 ▸), a similar approach has been used to express the multiplets of the final state (Ψ_*F*,*j*_) of a 2*p*–3*d* X-ray absorption spectroscopy (XAS) radiative process in terms of the corresponding excited-state crystal-field configurations,
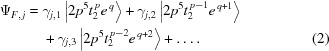
From this, the evaluation of the spin state and of the DOC, based on the contributions of charge-transfer configurations with respect to corresponding contributions of crystal-field-based configurations, has been successfully applied in several studies (Hocking *et al.*, 2006 ▸, 2007 ▸, 2009 ▸, 2010 ▸; Wasinger *et al.*, 2003 ▸).

*CTM4DOC* also calculates the metal-3*d* covalency for the ground state and performs a spectral deconvolution in terms of the various orbital contributions, based on the obtained projections for the ground-state and the final-state multiplets involved in the 2*p*–3*d* transition. Additionally, the program calculates Tanabe–Sugano diagrams for the multiplets in the ground and excited states with respect to variations of a given floating parameter (typically related to the crystal field), which is a useful tool for interpretation in optical, electron paramagnetic resonance and X-ray spectroscopies. A quick reference manual for *CTM4DOC* can be found in the supporting information 1.

## 2. Ground-state projections   

The single-point calculation of the ground state involves the unoccupied *d* shell of a transition metal, for which only the *F* ^2^ and *F* ^4^ Slater integrals and the *d*-spin orbit coupling constant are relevant atomic parameters. The *F* ^2^/*F* ^4^ Slater integrals describe the *dd*-interactions and they can be rewritten into the Racah *B* and *C* parameters (Griffith, 1961 ▸).

**Figure 3:**
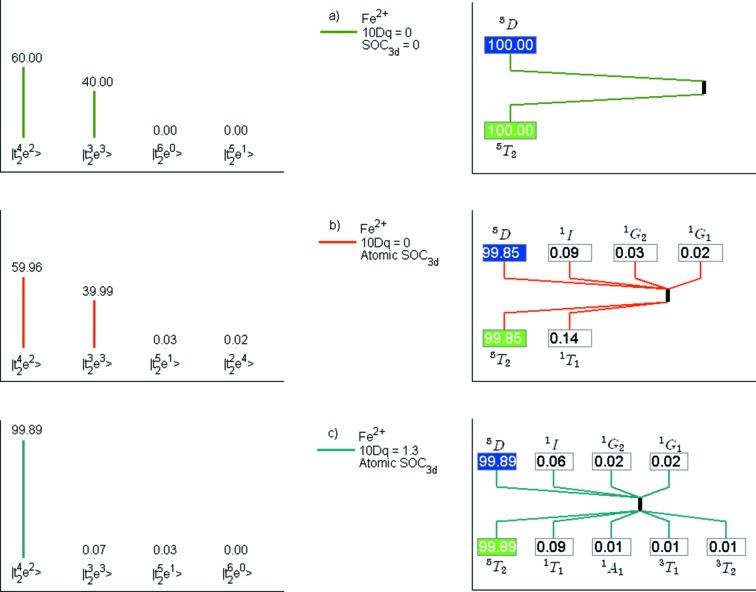
Ground-state projections in terms of crystal-field configurations (left); and cubic and atomic term symbols (right) for (*a*) atomic Fe^2+^ in the absence of 3*d* spin–orbit coupling; (*b*) atomic Fe^2+^; (*c*) and *O*_*h*_ Fe^2+^ (10*Dq* = 1.3 eV).

Fig. 3 ▸ shows the three projections implemented in *CTM4DOC* for Fe^2+^ in three different scenarios under *O*_*h*_ symmetry for the ground state. First, the valence spin–orbit coupling and the crystal-field are set to zero. The ground state is a linear combination of 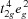 and 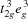 in 60:40 proportion, corresponding to 100% of the cubic term symbol ^5^ *T*_2_ and to the atomic term symbol ^5^ *D*. Then, when the valence (3*d*) spin–orbit coupling is set to its atomic value, the projection indicates a small amount of the crystal-field configurations 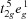 and 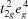 mixed in, corresponding to a small amount of singlet ^1^ *T*_1_. Finally, after turning on the crystal field (with a value of 10*Dq* = 1.3 eV), the projections indicate almost a pure crystal-field configuration, 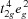, corresponding to the ^5^ *T*_2_ crystal-field term symbol and the ^5^ *D* atomic term symbol. An additional example for Co^2+^ is discussed in the supporting information.

**Figure 4:**
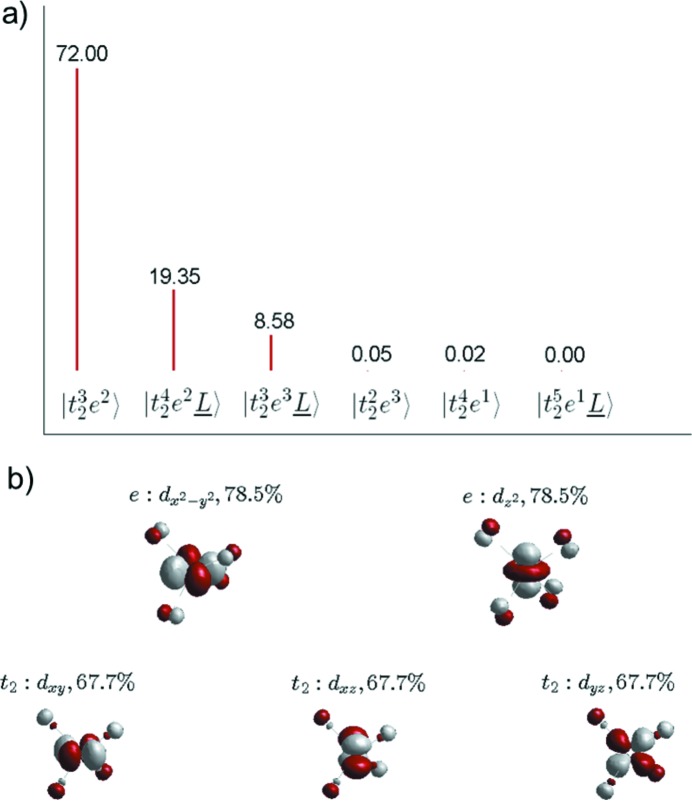
Ground-state projection in terms of crystal-field and LMCT configurations (*a*) and corresponding MO plots for Fe-3*d* based, *t*_2_ and *e* orbitals (*b*) for FeCl_4_^−^, reflecting the calculated DOC. The parameters used for the corresponding simulation are listed in Table 1 ▸.

To account for covalency, *CTM4DOC* can also model the crystal-field projection with charge-transfer configurations. In the current version of *CTM4DOC*, only ligand-to-metal charge-transfer (LMCT) parameters can be used, which implies that only the modelling of covalency for donor ligands is possible. In addition, only one charge-transfer state (*d* ^*N*+1^ *L*) is currently considered, which is sufficient to account for bonding in most molecular systems with only σ donor ligands. For other complexes, like in some solids, additional charge-transfer states (*e.g.* *d* ^*N*+2^ *L* ^2^) may be required and are not currently implemented. Thus, for charge-transfer calculations the ground state is expressed as an expansion of crystal-field configurations 3*d* ^*N*^ and of charge-transfer crystal-field configurations 3*d* ^*N*+1^ *L*,
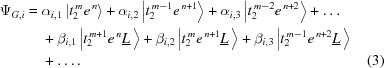
Fig. 4 ▸ shows an example for the ground state of FeCl_4_^−^ with parameters given in Table 1 ▸, under two different descriptions: (*a*) crystal-field configurations including LMCT and (*b*) the corresponding metal-3*d*-based molecular orbitals, revealing their orbital covalency, which could be potentially of great value for structure validation. In this regard, we propose that in combination with fitting of multiplet simulations to experimental data, metal-3*d* covalency can be extracted by performing a follow-up simulation in *CTM4DOC* for the ground state, using the fit parameters. We recognize that the fitting (manual or automatic) can lead to multiple solutions. Furthermore, these multiple fits may not only involve the uncertainties related to each multiplet simulation parameter but also include correlations between fit parameters. This implies that the method should involve the evaluation of metal covalencies with uncertainties derived from the fits. Nevertheless, the comparison of these empirically extracted covalencies from experimental data (*via* the fits) with those calculated by DFT can be used to validate structural models. This combined approach could be an alternative to *ab initio* calculations applied to spectroscopic techniques subject to multiplet effects where calculation times are prohibitive.

To further illustrate the method, we revisit here examples from a previous study (Wasinger *et al.*, 2003 ▸), where the experimental Fe *L*-edge XAS for a series of well characterized Fe complexes were manually fit with charge-transfer multiplet simulations; the fit parameters are then used to project the ground state into a linear combination of crystal-field configurations and to calculate the DOC; and finally the results are compared with DFT calculations. However, in the original study, the results obtained for the corresponding crystal-field projections are scaled according to the integrated intensity over the *L*-edge spectra. Here, we omit this step, as we realise now that the projection of the ground state should be independent of any observed XAS intensity. Remarkably, the metal-3*d* covalency obtained for *t*_2_ and *e* orbitals using *CTM4DOC* and the parameters given in Table 1 ▸ (from the manual *L*-edge XAS fits to experiment) are in excellent agreement with the covalency values obtained from their original DFT calculations (see Table 1 ▸). To explore the effects of changing the functional, we have performed new DFT calculations for the tetra­chloro and hexa­chloro iron complexes using BP86 and B3LYP. §S2 of the supporting information presents the results from these calculations and their comparison with the values given in Table 1 ▸.

## 3. Final-state projections   

In the current version of *CTM4DOC*, only the final state related to *L*-edge XAS in the absence of charge-transfer effects can be calculated. Moreover, only one of the descriptions in terms of crystal-field configurations [as in equation (2)] is available in this version of the program.

**Figure 5:**
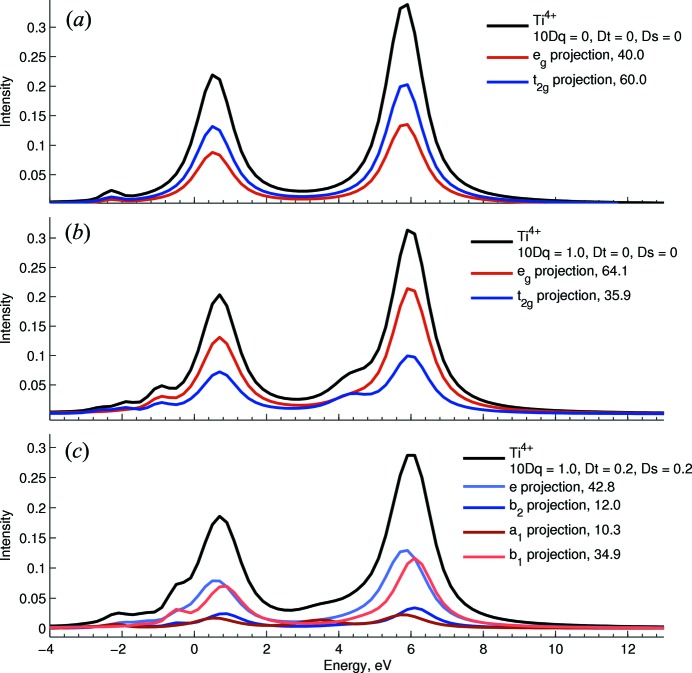
*L*-edge XAS spectra showing the projection of transitions into the different metal-3*d*-based orbitals for Ti^4+^ systems under (*a*) atomic, (*b*) octahedral and (*c*) *D*_4*h*_ symmetries. The *t*_2_:*e* intensity ratio becomes closer to expected values (based on 3*d*-occupation) as the symmetry is reduced (see text).

**Figure 6:**
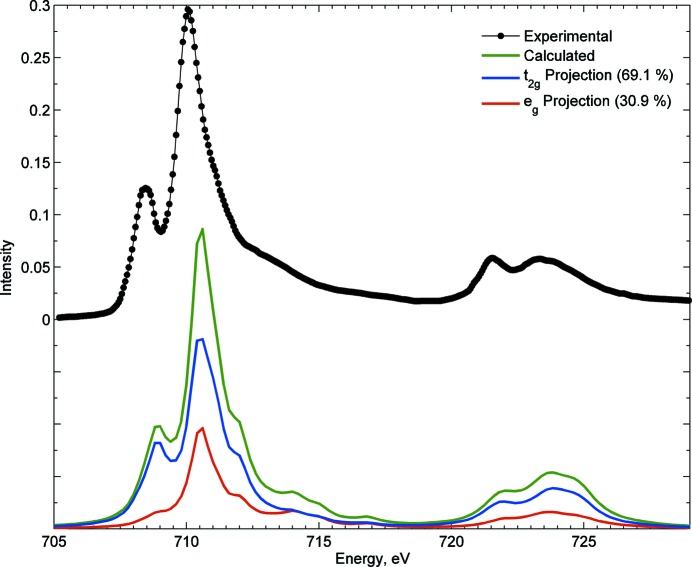
Experimental *L*-edge XAS spectrum (de Groot *et al.*, 2005 ▸) compared with calculation and with corresponding projections of transitions into metal-3*d* based orbitals of *t*_2_ and *e* symmetries in an octahedral Fe^3+^ complex (10*Dq* = 1.5 eV and 90% of reduction in the atomic values of Slater integrals). Contrary to Ti^4+^ and based on the large number of multiplets in the case of Fe^3+^, the *t*_2_:*e* proportion in this case is closer to the expected 3:2 value (based on 3*d*-occupation).

In the current version we have implemented a projection for *L*-edge XAS which shows the total spectrum and a deconvolution in terms of specific transitions to *t*_2_ and *e* orbitals in cubic symmetry (or the corresponding orbitals in *D*_4*h*_ symmetry). This is useful to interpret the spectrum in terms of a single-particle model. To accomplish this, each of the multiplets that are allowed in the final state are expanded according to equation (2), and the corresponding ground-state multiplet expanded according to equation (1). Then, the dipole transition integral for each *j* transition is expressed in terms of such expansions to obtain equation (4),
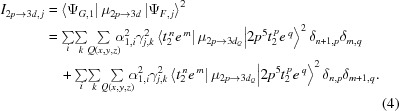
We note here that any term involving a transition that changes any occupation number by more than 1 is zero. This does not mean that two-electron processes are not occurring in *L*-edge XAS. Instead, equation (4) explicitly reveals the mechanism from which such transitions are possible, under an orbital description of the involved electronic states. We also note that this description holds under the consideration that any one-electron transition, which essentially involves putting a 2*p* electron into a *t*_2_ orbital [first term of equation (4)] or into an *e* orbital [second term of equation (4)], has the same 2*p*-to-3*d* oscillator strength (integrated over all *x, y* and *z* directions and averaged over all 2*p* donor orbitals relevant to each component). Then it follows that the intensity projection for each *j* transition in an *L*-edge XAS spectrum, which corresponds to transitions to *t*_2_ or *e* orbitals, is given by equations (5) and (6), respectively,
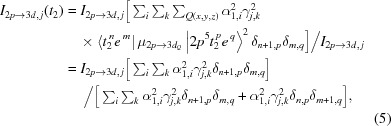


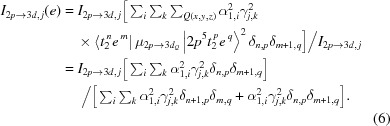
Moreover, an interesting observation, which was first apparent in a recent study (Kroll *et al.*, 2015 ▸) focusing on demonstrating the projection of the final state in the *L*-edge XAS of Ti^4+^, is that in octahedral symmetry, out of the 25 multiplets emerging from the 2*p* ^5^3*d* ^1^ final state, which collectively carry the expected 6:4 ratio (consistent with the ratio of *t*_2_:*e* holes in a 3*d* ^0^ ground state), only seven are allowed by the electric dipole in *L*-edge XAS, which yields a different *t*_2_:*e* ratio than the one expected based on the number of holes (see Fig. 5*b* ▸). In other words, the electric dipole operator does not allow transitions to *t*_2_ and *e* states *in the same proportion under cubic symmetry*, and overall there is a preference for *e* states, likely related to the fact that the relative orientation of the dipole components +1, −1 and 0 of light being parallel to the orientation of orbitals with *e* symmetry (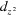 and 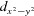), and perpendicular to orbitals with *t*_2_ symmetry. Further, equation (4) provides a nice way to visualize also how multiplet effects promote the mixing of configurations which causes the loss of *t*_2_ character, again under the assumption that the oscillator strength for individual 2*p* to 3*d* transitions (regardless of the *d*-symmetry) is constant. We note from Fig. 5(*a*) ▸ that, under spherical symmetry (atomic) for *d* ^0^ systems, each multiplet in the final state is composed of exactly 60% 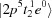 and 40% 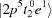 which preserves the expected *t*_2_:*e* ratio. We also observe a recovery of this *t*_2_:*e* intensity ratio as the number of multiplets increase in the final state, like in the case of Fe^3+^ (see Fig. 6 ▸) in comparison with Ti^4+^. The same effect is observed when the symmetry is further lowered, for example from *O*_*h*_ to *D*_4*h*_, as shown in Fig. 5(*c*) ▸ where the ratio of *e* + *b*_2_ (collectively *t*_2_ in *O*_*h*_) and *a*_1_ + *b*_1_ (collectively *e* in *O*_*h*_) intensities of ∼55:45 start approaching a 60:40 ratio.

We anticipated also that this ratio should be close to the expected *t*_2_:*e* ratio (based on number of holes) in second- and third-row transition metals whose *L*-edge XAS spectra become dominated by 2*p* spin–orbit coupling and do not affect the mixing of *d*-related states. However, the calculation of *L*-edge XAS of second- and third-row transition metals will only be available in future versions of *CTM4DOC*. Also, future versions of the program will include the projections in the final state that include LMCT and MLCT states. This will allow for the details on how the *t*_2_:*e* ratio is effectively changed by LMCT and for the decomposition into the additional back-bonding orbitals that come into play for MLCT calculations.

## 4. Tanabe–Sugano and single-point energy diagrams   

The projections described in the previous sections, as implemented in *CTM4DOC*, are not only performed for the ground state but also for the rest of the multiplets within an initial- or a final-state configuration. The graphical user interface of *CTM4DOC* allows the visualization of these additional projections by choosing a different multiplet (other than the ground state) *via* an energy selector. Conveniently, an energy diagram displaying all multiplets is also calculated. Further, to explore the effect of one of the parameters on the energy and nature, in terms of the above-discussed electronic structure descriptions, of all multiplets, *CTM4DOC* extends the same calculations to reproduce Tanabe–Sugano diagrams, from which individual energy diagrams and specific projections to a given state can be extracted and displayed.

**Figure 7:**
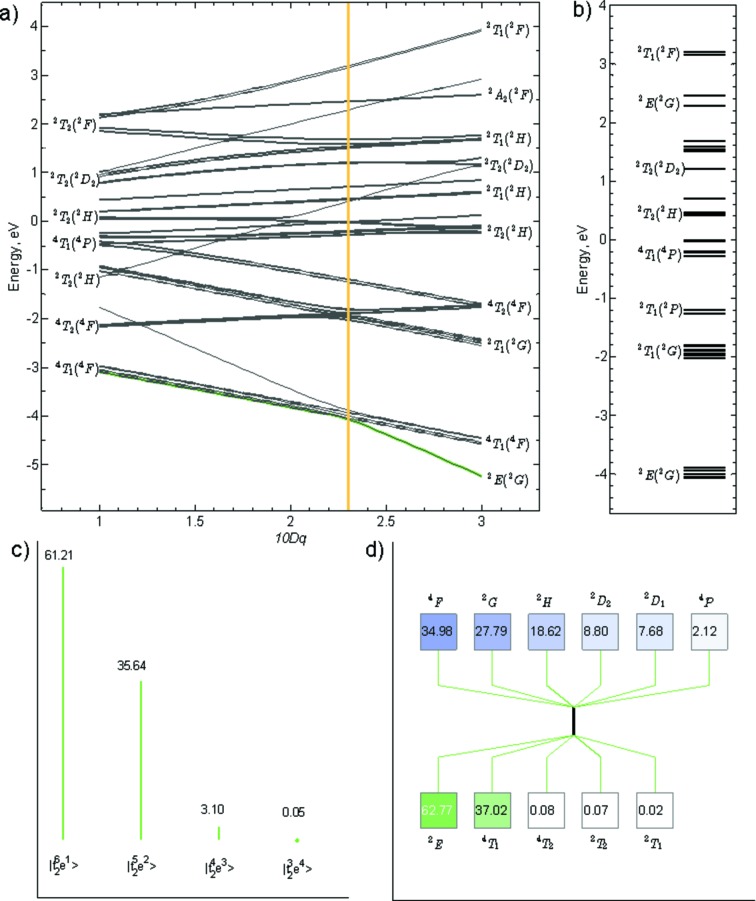
(*a*) Tanabe–Sugano diagram for Co^2+^ with a floating octahedral field ranging from 1 to 3 eV. No reduction in the Slater integrals is used. (*b*) Energy diagram at 10*Dq* = 2.3 eV, which is close to the crossover from high to low spin. Corresponding projection in terms of (*c*) crystal-field configurations and (*d*) atomic and cubic term symbols.

This is potentially useful in many different types of spectroscopies. We emphasize here its applicability to soft X-ray resonant inelastic X-ray scattering (RIXS), where a quick comparison of the RIXS peaks with a ground-state Tanabe–Sugano diagram can reveal crystal-field and charge-transfer parameters (when applicable). In addition, many systems can exist in a state of spin admixture, which cannot be accurately described in terms of a single spin-state configuration. A Tanabe–Sugano diagram helps reveal the conditions under which such situations may arise and assists in providing detailed electronic structure descriptions. For instance, Fig. 7(*a*) ▸ shows a Tanabe–Sugano diagram for a Co^2+^ *O*_*h*_ complex in the range of energy for 10*Dq* of 1–3 eV and with Slater integrals taken at their atomic value. Then, the energy diagram of Fig. 7(*b*) ▸ corresponds to the cut shown in the Tanabe–Sugano near the crossing point (at 2.3 eV) and so are the projections shown for the ground state (highlighted in the energy diagram) in Figs. 7(*c*) and 7(*d*) ▸, corresponding to a crystal-field configuration description; and a cubic and an atomic term symbol descriptions, respectively.

We note that at a 10*Dq* of 2.3 eV, with Slater integrals and 3*d* spin–orbit coupling taking their atomic values, the ground state is a mixture of spin states. It consists, first, of 63% of the cubic doublet ^2^ *E*, originating from a mixture of mainly the atomic term symbols ^2^ *G*, ^2^ *H*, ^2^ *D*_1_ and ^2^ *D*_2_; and, second, of essentially 37% of the quartet ^4^ *T*_1_, originating from the 35% of the atomic term symbol ^4^ *F* and 2% of the atomic term symbol ^4^ *P*. This mixture of spin states is also apparent from the crystal-field description of Fig. 7(*c*) ▸, which correspondingly reveals 61% of the crystal-field configuration 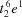, 36% of the crystal-field configuration 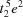 and 3% of the crystal-field configuration 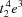. An additional Tanabe–Sugano diagram example for Co^3+^ is discussed in the supporting information.

## 5. Conclusions   

In the present manuscript we have introduced *CTM4DOC*, a Matlab-based program to perform electronic structure calculations for the analysis and interpretation of X-ray spectra. Two electronic structure descriptions, one based on one-electron orbitals and a second one based on multi-electron atomic and cubic term symbols, have been implemented. The program is equipped with a graphical user interface that allows the user to explore the evolution of detailed electronic state descriptions in the ground and excited states of transition metal systems subject to changes in the atomic, crystal-field and charge-transfer parameters. Changes in the energies of the electronic states, as crystal-field parameters are changed, can be tracked and summarized as Tanabe–Sugano diagrams.

Several examples in transition metal systems have been presented to illustrate the different features of the program. Furthermore, we demonstrate how electronic systems that are often thought to be simply low-spin or high-spin are really of mixed-spin nature. This observation could lead to more accurate interpretations of experimental data and better rationale of physical and chemical properties. Moreover, we believe that *CTM4DOC* is a valuable tool to determine covalency from experiment; first, the set of parameters that best replicates experimental X-ray data is determined and then a DOC calculation is performed using these parameters. This combined approach can also be very useful for structure validation when comparing the results directly with DFT calculations, especially for highly correlated systems where *ab initio* methods aimed to reproduce the experimental data are computationally expensive.

## 6. Related literature   

The following references are mentioned in the supporting information: Becke (1988 ▸); Delgado-Jaime & DeBeer (2012 ▸); Eichkorn *et al.* (1995 ▸); Eichkorn *et al.* (1997 ▸); Grimme (2004 ▸); Iikura *et al.* (2001 ▸); Klamt & Schüürmann (1993 ▸); Neese (2012 ▸); Pantazis *et al.* (2008 ▸); Perdew (1986 ▸); Szabo & Ostlund (1989 ▸); Weigend (2006 ▸); Yanai *et al.* (2004 ▸).

## Supplementary Material

Supporitng text, figures and tables. DOI: 10.1107/S1600577516012443/hf5317sup1.pdf

## Figures and Tables

**Table 1:** List of parameters used in the multiplet simulations for the extraction of metal-3*d* orbital covalencies of *t*_2_ and *e* Fe-3*d*-based orbitals in compounds 1–9 (Wasinger *et al.*, 2003 ▸); for comparison, the corresponding DFT-calculated covalencies (Wasinger *et al.*, 2003 ▸) are also listed

					Metal-3*d* covalency (%)		DFT (BP86) covalency (%)	
Complex	10*Dq*	Delta	*T*(*t*_2_)	*T*(*e*)	*t*_2_	*e*	*t*_2_	*e*
1-[FeCl_4_]^−^	−0.5	0.1	1.5	1.1	68	78	68	77
2-[FeCl_6_]^3−^	1.2	0.1	0.9	1.75	83	61	85	64
3-[FeCl_4_]^2−^	−0.3	3.0	1.45	1.45	83	78	84	89
4-[FeCl_6_]^4−^	0.6	1.75	0.45	0.9	94	85	94	83
5-Fe(acac)_3_	1.5	0.8	0.9	1.7	84	69	83	68
6-[Fe(ox)_3_]^3−^	1.5	0.8	0.9	1.6	84	71	83	72
7-[Fe(ida)_2_]^−^	1.6	0.6	0.9	1.7	83	68	83	68
8-[Fe(tacn)_2_]^3+^	2.2	2.8	0.9	3.4	95	55	93	62
9-[Fe(tacn)_2_]^2+^	1.7	2.8	0.3	1.8		73		71

## Footnotes

1 A quick reference manual for *CTM4DOC* is provided. Along with it, the file CTM4DOC_v.1_examples.mat with all examples discussed here is also included. To visualize and explore this file, open *via* ‘Load Session’ in *CTM4DOC*. *CTM4DOC* is directly available through our website. Additionally, §S2 presents the results from new DFT calculations performed for iron chloride complexes of Table 1 ▸ using two different functionals, BP86 and B3LYP.
