# Supplementary material for: CTM4DOC: electronic structure analysis from X-ray spectroscopy
Source: J Synchrotron Radiat. 2016 Aug 23;23(Pt 5):1264–71. doi: 10.1107/S1600577516012443 (PMC5006656; doi:10.1107/S1600577516012443)
Supplement: Supplementary file 1 [file s-23-01264-sup1.pdf]

JOURNAL OF
SYNCHROTRON
RADIATION

Volume 23 (2016)

Supporting information for article:

CTM4DOC: Electronic structure analysis from X-Ray Spectroscopy

Mario Ulises Delgado-Jaime, Kaili Zhang, Josh Vura-Weis and Frank M. F. de Groot

S1. Quick Reference Manual for CTM4DOC

S1.1. Getting Started

The current version of the program is only available for Windows (64 bit systems.) The program cowan must be installed in C : (if you are already a user of CTM4XAS, this program is already installed). In addition, the proper MCRInstaller executable must be also installed before first-time execution of CTM4DOC (visit http://nl.mathworks.com/products/compiler/mcr/ and download the R2015a version for Windows 64 bit, which is the Matlab version used to compile CTM4DOC v1.0.) Running the file *MyAppInstaller_web.exe* should retrieve the correct MCR file from Internet. Note that future versions of the program may require a different MCRInstaller file.

To run CTM4DOC, double click on CTM4DOC.exe. A *cmd* console window and a graphical user interface, such as in Figure 2 will appear.

All the examples discussed in this manuscript are contained in the accompanying file CTM4DOC_v1.0_examples.mat, which can be loaded to CTM4DOC through 'File' and 'Load Session'.

Running new jobs append to the current list and may produce the following files which can be further reused by proficient users of cowan : rcg, rac, org, ora, ban, oba, _LS.ban, and/or _LS.oba.

S1.2. Atomic System

In the current version of CTM4DOC, only calculations on first-row transition metal systems, in addition to calculations on K^{+} and Ca^{2+} systems, are possible. The full list of systems available can be displayed by clicking on the search ('…') button. Alternatively, one can modify directly the system in the edit field, the syntax being of the form M*x*+, where M = metallic system and *x*+ = oxidation state (e.g., K1+, Ni2+, Ti4+, etc.)

All ground-state calculations include a LMCT configuration and for the moment this cannot be disabled (see section S1.5.). Upon selecting the atomic system, the relevant configurations get updated.

S1.3. Atomic Parameters

Slater integrals and spin-orbit coupling parameters can be scaled down and up from their atomic value. By default, the atomic value is taken at the 80 % of the Hartree-Fock atomic values derived from Cowan RCN calculations. Ground-state calculations require only scaling F_{dd} Slater integrals and valence (3d) spin-orbit coupling values. Final-State calculations require additional scaling values of F_{pd}, G_{pd} Slater integrals and of core (2p or 3p) spin-orbit coupling values.

S1.4. Crystal-Field Parameters

Calculations for O_h, T_d (negative 10Dq) and D_{4h} systems are available in this version of CTM4DOC. More symmetries will be added in future versions.

S1.5. Charge-Transfer Parameters

In the current version of CTM4DOC, only LMCT configurations are available and only for ground-state calculations. This means that only the covalency for donor ligands can be evaluated at this time. More options are to be added in future releases. The default values of LMCT parameters are such that the mixing of configurations is not allowed. The hoping parameters $t(t_2)$ and t(e) in O_h-T_d symmetry (or t(e), $t(b_2)$, $t(a_1)$ and $t(b_1)$ in D_{4h} symmetry) control the extent to which the mixing of LMCT occurs. If no LMCT is required for a ground-state calculation, simply use the defaults and ignore states that are close to 10 eV.

S1.6. Projection Options

For ground-state calculations, both crystal-field (O_h-T_d and D_{4h}) and atomic/cubic term symbol projections are available. For final-state calculations only crystal-field projections are for the moment possible. Only final-state calculations related to L-edge XAS spectroscopy are currently available. Tanabe-Sugano diagrams can be constructed by floating any atomic and crystal-field parameters. In ground-state calculations, the Δ (delta) parameter of the LMCT configuration can also be floated.

S1.7. Plotting Results

CTM4DOC present the outputs in various forms that can be selected from the menu located at the lower left corner. A brief description is given below for each option. We encourage the reader to use the examples in the file “CTM4DOC_v1.0_examples.mat” to explore and get familiar with these visualizations. Note that the options “Crystal-field projection”, “Energy diagram”, “Spectrum”, and “Atomic/Cubic term-symbol projection” get affected by the ‘Stack’ check box. When this check box is active, all jobs that include selected option are displayed simultaneously. Thus, to visualize them individually, the reader should uncheck the ‘Stack’ box.

Crystal-field projection – Describes the selected electronic state in terms of the six highest crystal-field contributors in the expansion given by equation 3 (ground-state calculations) or equation 2 (final-state calculations.) Figures 3, 4a and 7c provide examples of this.

Energy diagram – Shows the energy diagram of all electronic states relative to an average energy of 0 eV. If the toggle button labelled ‘Zero Ground’ is active, then the energy of the states are relative to the lowest-energy state displayed. The selected electronic state (from the “State #” menu) always appears highlighted. For final-state calculations, the energy diagram also highlights, by overlaying the

corresponding spectrum, which multiplets in the final-state are accessible by the corresponding radiative process (L-edge XAS in this case.)

Spectrum – In final-state calculations, it shows the corresponding spectrum (only L-edge XAS for the current version) and the projection of transitions into orbitals, like in the cases shown in Figures 5 and 6. Moreover, the user can change the broadening appearance of the spectrum and the corresponding projections by changing the parameters located in the bottom part of the interface. A numerical convolution is performed using the provided Gaussian width (fwhm in eV) and a modulated Lorentzian width (fwhm in eV), which changes from a minimum to a maximum value in the following way: before a given pivot energy, the Lorentzian width is the minimum value. Then, starting at this pivot energy, the Lorentzian width increases until reaching a maximum value at an energy equal to the pivot energy plus the energy gap. Then, at higher values of that energy, the Lorentzian width is given simply by the maximum value. For example, the behavior of the Lorentzian width in L-edge XAS of first-row transition metals is such that the gap parameter need to be set as zero and the pivot energy can be set anywhere between the L_2 and L_3 edges. Other behaviors are expected, however, in K-edge XAS and Kβ XES spectroscopies, both of which are to be included in future releases of CTM4DOC.

Tanabe-Sugano diagram – Shows the effect of one floating parameter in the energy of the multiplets, and are useful in the interpretation of UV-Vis and soft-x-ray RIXS spectra of transition metals ions. In addition, it highlights regions where more than one configuration is contributing to the ground-state and where this leads to a mixed spin state. From the last example in the file "CTM4DOC_v1.0_examples.mat", the reader can display the corresponding Tanabe-Sugano diagram for Co^{3+} (see Figure S1.) In this case, between a 10Dq of 2.1- 2.2 eV we observe mixing of spin-states. Many Co^{3+} complexes can easily fall into this range, which widens when including LMCT effects. Under LMCT, the range of 10Dq itself moves to lower values, which increases the number of Co^{3+} systems that could be classified as mixed spin-spin systems.

Differential orbital covalency – Shows the MO plots expected for the metal -3d based molecular orbitals reflecting the covalency that is consistent with the calculated expansion of crystal-field + LMCT configurations in the ground-state (equation 3.) For systems in which more than one $3d^n$ base-configuration has a significant contribution, the calculation of covalency is performed using only the major contributor as an approximation. Strictly speaking, though, this molecular orbital picture breaks down in systems where low-lying excited state configurations are mixed in.

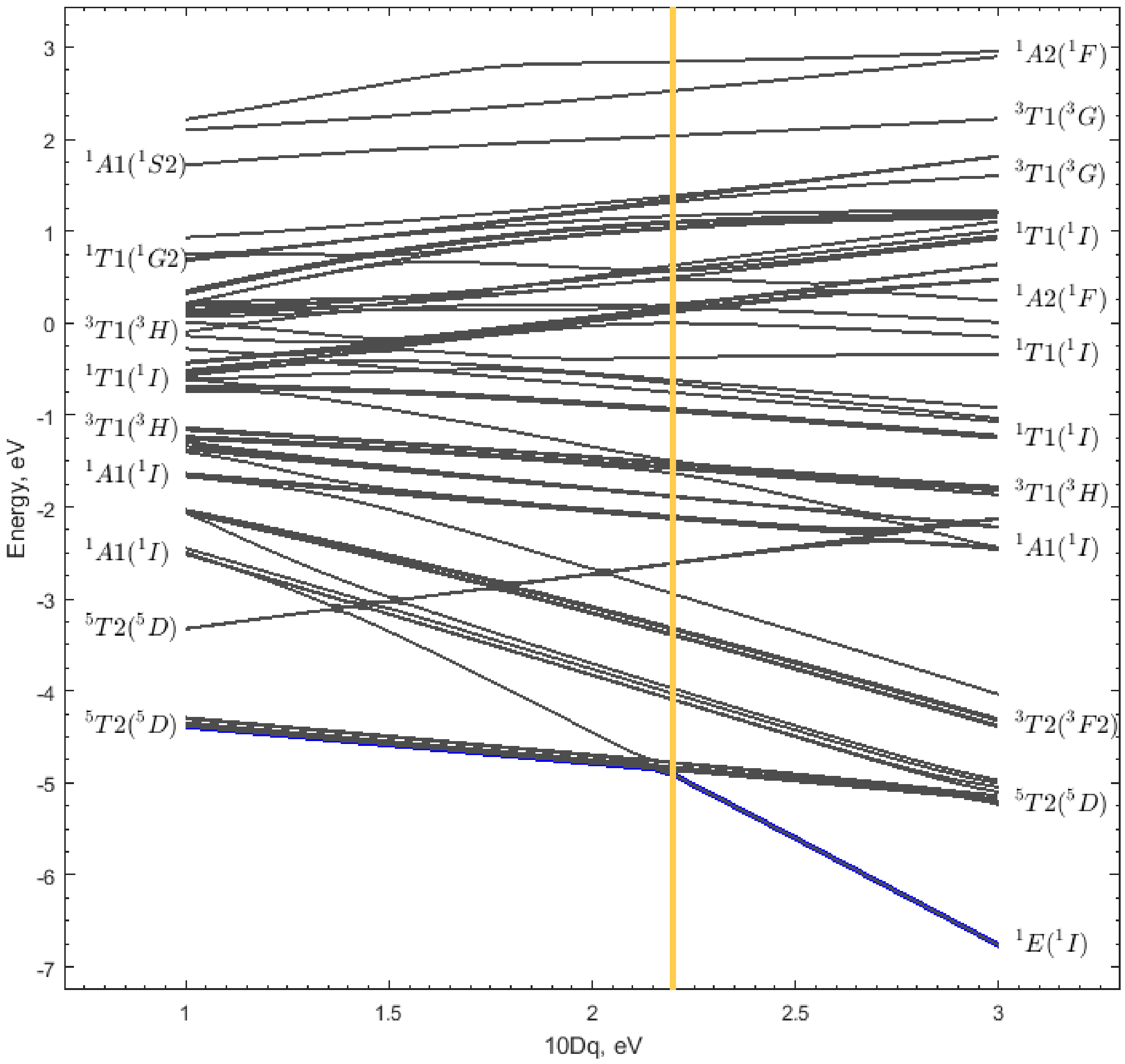

Figure S1 Tanabe-Sugano diagram for O_h Co^{3+} highlighting the ground-state (blue line) and the floating point corresponding to a 10Dq of 2.2 eV (orange line.) The simulations were performed using no reduction in Slater integrals and no reduction in 3d spin-orbit coupling.

Atomic/cubic term-symbol projection – Displays the expansion of the selected multiplet in terms of the atomic and cubic term-symbols.

Crystal-field projection composition diagram – This is a one-dimensional phase diagram available only for Tanabe-Sugano calculations which shows the overall top five contributors of the crystal-field expansion of equation 1 in the ground-state at every point of the floating variable. Figure S2 shows the diagram corresponding to the Tanabe-Sugano diagram displayed in Figure S1.

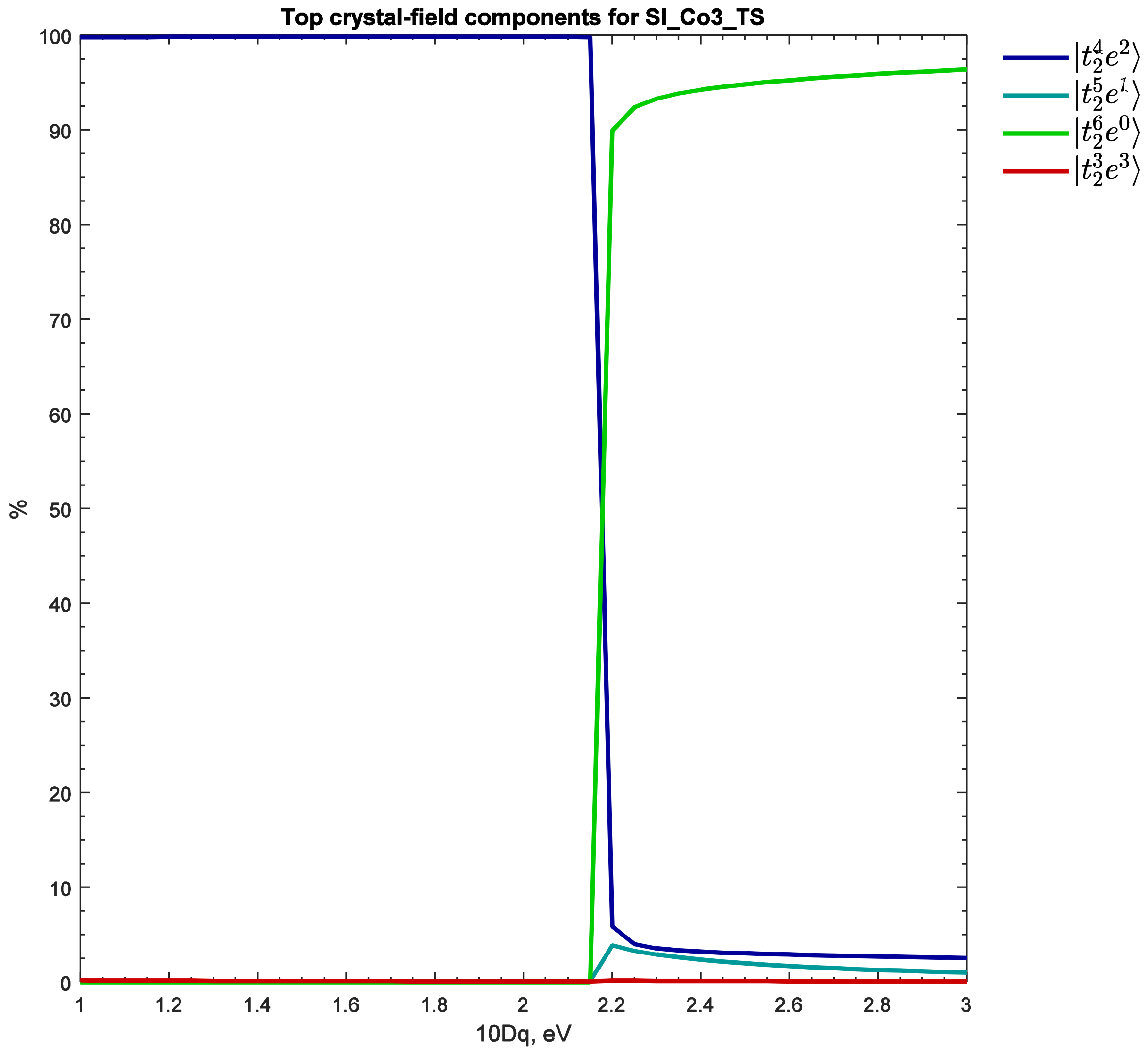

Figure S2 Composition diagram for the Tanabe-Sugano diagram given in Figure S1 in terms of crystal-field projection components.

Atomic term-symbol projection diagram - This is a one-dimensional phase diagram that shows the overall top five atomic-term symbols in a Tanabe-Sugano type of calculation. Figure S3 shows an example for the Tanabe-Sugano diagram shown in Figure S1.

Cubic term-symbol projection diagram – Like in the case of the atomic term-symbol projection diagram (see above), this is a one-dimensional phase diagram that displays the overall top five cubic term-symbols in a Tanabe-Sugano calculation. Figure S4 shows an example corresponding to the Tanabe-Sugano diagram of Figure S1.

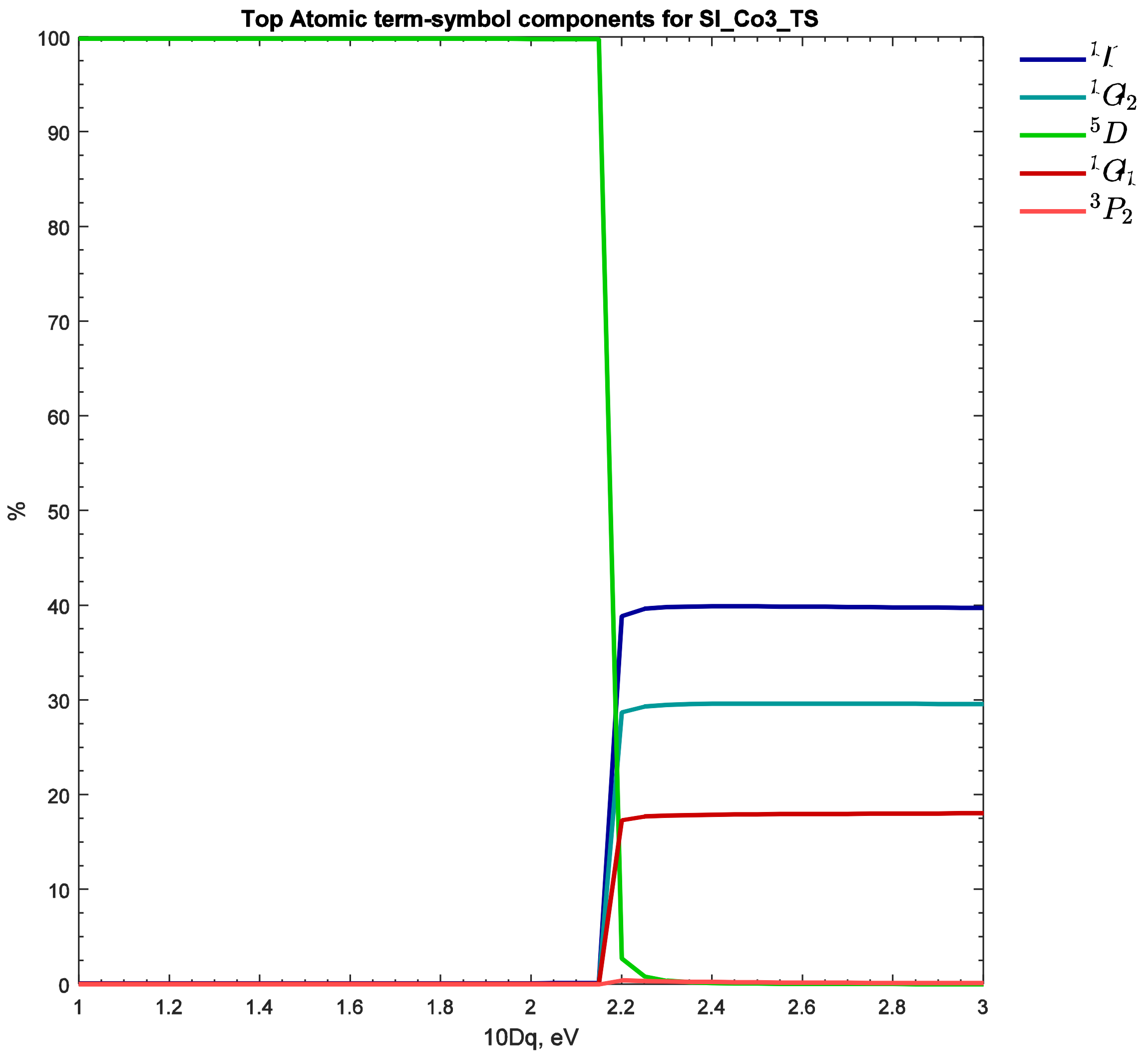

Figure S3 Composition diagram for the Tanabe-Sugano diagram given in Figure S1 in terms of major atomic term symbols components.

S1.8. Exporting Results

Every figure can be edited and then saved in several figure formats. Alternatively, every job can be exported as an output file, which prints all calculated projections for all calculated multiplets in the selected job. This option is available through ‘File” and ‘Export’.

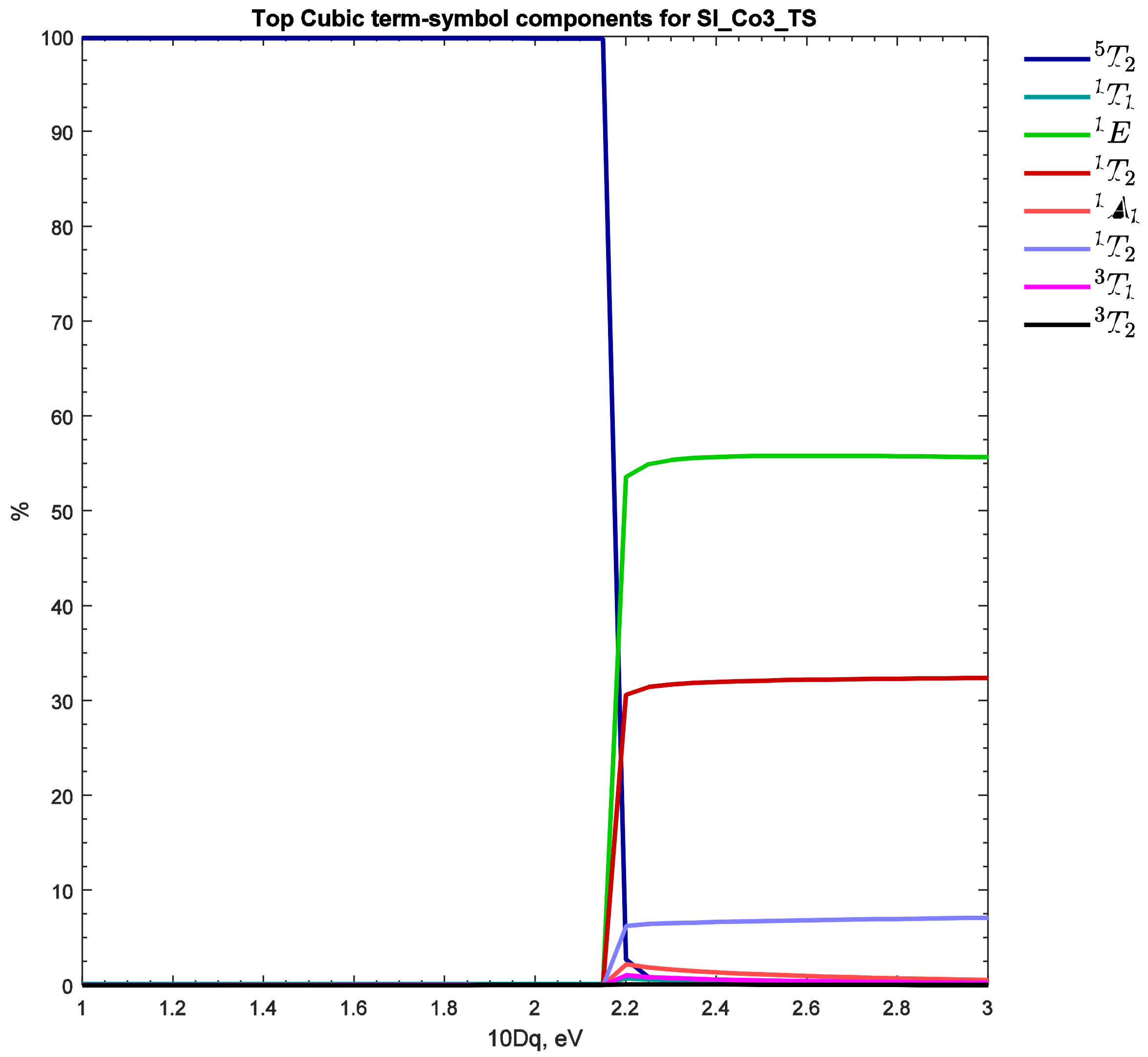

Figure S4 Composition diagram for the Tanabe-Sugano diagram given in Figure S1 in terms of major cubic term-symbol components.

S2. Additional DFT calculations in iron chlorides

All electronic structure calculations were performed using the ORCA quantum chemistry package (version 2.9).[1] Unconstrained geometry optimizations and frequency calculations of all compounds were carried out at the DFT level, using either the BP86[2] or the B3LYP[3] functionals. Initial geometries were idealized T_d (for tetrachloro complexes) or O_h (hexachloro complexes) with Fe-Cl bond distances of 2.19 Å (in the case of Fe^{3+}) and of 2.25 Å (in the case of Fe^{2+}). The segmented all-electron relativistically contracted (SARC) def2-TZVP(-f) basis set of triple−ζ quality was used on all atoms, combined with the corresponding def2-TZV/J auxiliary basis.[4] The radial integration accuracy on Fe was increased to 7 (SpecialGridIntAcc 7.) Moreover, to account for charges, the complexes were embedded in a conductor-like screening model (COSMO) with an infinite dielectric in all calculations.[5] In the optimized structures and corresponding ground-state wave function,

MOAnalyzer[6] was used for fragment population analysis based on the calculated Loewdin Population Analysis.[7] The covalency values reported in Table S.1 below were then directly extracted from the fragment population analysis of the unoccupied Fe-3d based molecular orbitals.

Table S1 Comparison of covalency values given in Table 1 with DFT calculations based on new calculations as described above.

	Covalency Experimental[a]		DFT BP86[b]		BP86[c]			B3LYP[c]		
	t_2	e	t_2	e	t_2[d]		e	t_2[d]		e
Complex					(M-d)	(M-p)		(M-d)	(M-p)	
$[FeCl_4]^-$	68	79	68	77	69	3	80	71	3	84
$[FeCl_6]^{3-}$	83	61	85	64	86	-	63	89	-	64
$[FeCl_4]^{2-}$	83	79	84	89	82	3	90	82	5	92
$[FeCl_6]^{4-}$	94	85	94	83	93	-	81	92	-	84

a – Obtained from simulations in CTM4DOC from crystal-field + LMCT parameters obtained from manual fits to experimental data, as given in Table 2 of reference 13.

b- Obtained from original DFT calculation in reference 13 performed in ADF on optimized models.

c- Obtained from new DFT calculations.

d- For T_d complexes, the breakdown of t_2 orbitals into the metal-p and metal-d contributions is also given.

S3. References

[1] F. Neese, *WIREs Comput. Mol. Sci.* **2012**, *2*, 73-78.

[2] a) A. D. Becke, *Physical Review A* **1988**, *38*, 3098-3100; b) J. P. Perdew, *Physical Review B* **1986**, *33*, 8822-8824.

[3] a) S. Grimme. *J. Comput. Chem.*, **2004**, 25, 1463; **b)** H. Iikura, T. Tsuneda, T. Yanai, and K. Hirao. *J. Chem. Phys.*, **2001**, *115*, 3540; **c)** T. Yanai, D. P. Tew, and N. C. Handy. *Chem. Phys. Lett.*, **2004**, *393*, 51.

[4] a) D. A. Pantazis, X. Y. Chen, C. R. Landis and F. Neese, *Journal of Chemical Theory and Computation* **2008**, *4*, 908-919; b) F. Weigend, *Physical Chemistry Chemical Physics* **2006**, *8*, 1057-1065; c) K. Eichkorn, O. Treutler, H. Ohm, M. Haser and R. Ahlrichs, *Chemical Physics Letters* **1995**, *240*, 283-289; d) K. Eichkorn, F. Weigend, O. Treutler and R. Ahlrichs, *Theoretical Chemistry Accounts* **1997**, *97*, 119-124.

[5] A. Klamt and G. Schuurmann, *Journal of the Chemical Society-Perkin Transactions 2* **1993**, 799-805.

[6] M. U. Delgado-Jaime and S. DeBeer, *Journal of Computational Chemistry* **2012**, *33*, 2180-2185.

[7] A. Szabo and N. S. Ostlund. *Modern Quantum Chemistry: Introduction to Advanced Electronic Structure Theory*. Dover Publications, 1989.
